# Persistence of *Candida dubliniensis* and lung function in patients with cystic fibrosis

**DOI:** 10.1186/s13104-017-2656-z

**Published:** 2017-07-26

**Authors:** Atqah AbdulWahab, Husam Salah, Prem Chandra, Saad J. Taj-Aldeen

**Affiliations:** 1 Departments of Pediatrics, Hamad Medical Corporation, Doha, Qatar; 2 Weill Cornell Medicine, Doha, Qatar; 3 Microbiology Division, Laboratory of Medicine and Pathology, Hamad Medical Corporation, Doha, Qatar; 4 CBS Fungal Biodiversity Center, Utrecht, The Netherlands; 5 Department of Medical Research, Hamad Medical Corporation, Doha, Qatar

**Keywords:** *Candida dubliniensis*, Cystic fibrosis, Lung function, BMI, FEV1%

## Abstract

**Objectives:**

*Candida dubliniensis* is an emerging yeast and demonstrated a high adherence property to cystic fibrosis respiratory tract. Therefore, it is important to determine the persistence of *C*. *dubliniensis* and to assess the possible relationship to the body mass index (BMI) and forced expiratory volume in 1st second (FEV1).

**Results:**

*Candida* isolates were identified by MALDI-TOF MS to species level from 40/52 (76.9%) cystic fibrosis patients. *C. dubliniensis* was the most common organism isolated from 50/77 (65%) lower respiratory specimens of 29 patients. Patients with persistent *C. dubliniensis* isolates have higher mean BMI in comparison to intermittent *C. dubliniensis* group. However, this difference did not reach statistical significance (P = 0.539). In contrast, patients with persistent *C. dubliniensis* isolates have significantly lower FEV1% mean in comparison to intermittent *C. dubliniensis* group particularly at initial two visits (P < 0.05); however, at subsequent visit the difference observed was not statistically significant (P = 0.456). The persistence of *C. dubliniensis* is more frequent in adults having more advanced disease, co-infections with chronic *P. aeruginosa*, cystic fibrosis related diabetes, long-term nebulized tobramycin and oral Zithromax therapy than patients with intermittent *C. dubliniensis.* Patients with persistent *C. dubliniensis* have lower FEV1 percentage and higher BMI than the intermittent *C. dubliniensis*.

## Introduction

Improved management of airway infections and airway clearance in cystic fibrosis (CF) has resulted in the emergence of new pathogens and intrinsically resistant to a broad spectrum of antibiotics [1, 2]. The incidence and diversity of fungi isolated from the respiratory secretion of CF patients are increasing. Among clinically significant fungi, *Candida* spp. are the most common yeasts, whereas *Aspergillus* spp., *Scedosporium apiospermum*, as well as *Exophiala dermatitidis,* were reported the most common molds recovered from respiratory secretions of CF patients [3, 4]. The isolation rate of fungi varies considerably according to different studies [5]. One fungal genus isolated at high frequencies from sputum culture of CF patients is *Candida* with *C. albicans* being reported as the most common yeast with the highest prevalence rate of up to 87.9% [5–7]. *C. dubliniensis* has emerged over the last decade in individuals with candidemia, CF patients, and both HIV and non-HIV patients [8–12], and is the second yeast isolated from respiratory samples of CF patients after *C. albicans* and in some studies after *C. parapsilosis* [12, 13].

Recently, we reported a high frequency of *C. dubliniensis* reaching up to 68% from the lower respiratory tract of CF patients and demonstrated a high adherence property [14]. Association has been reported between nutritional status measurements, the body mass index (BMI) and pulmonary function were assessed using spirometry, using forced expiratory volume in 1st second (FEV1) to classify the severity of airway obstruction due to the lower respiratory infections in children and adolescents with CF [15]. Therefore, it is worth to determine the persistence of *C*. *dubliniensis* in the lower respiratory samples of Pediatric and adult CF patients and to assess the possible relationship to the body mass index (BMI) and forced expiratory volume in 1st second (FEV1).

## Main text

### Methods

#### Study design and patients

A prospective observational study of both pediatric (≤18 years) and adult (>18 years) CF patients over a period of 14 months was carried out at Hamad Medical Corporation in the State of Qatar between March 2013 and May 2014. The routine CF clinic visit in our center was followed once every 3–4 months’ interval in which anthropometric measurements, lower respiratory samples, and pulmonary function were recorded at each clinic visit. The inclusion criteria that each CF patient has at least two lower respiratory secretions either from an outpatient or in-patient setting with an interval 2–5 months between specimens. The study was approved by the Qatar Foundation Proposal Number NPRP9-094-3-017 and the research ethics committee or institutional review board (IRB) (proposal 16149 Medical Research Center, Hamad Medical Corporation).

#### Data collection

A number of demographic, anthropometric, clinical and other parameters such as patient’s age, gender, BMI, cystic fibrosis transmembrane regulator (CFTR) genotype, the presence of pancreatic insufficiency, the occurrence of CF-related diabetes and bacterial isolates were recorded in each clinic visit or during admission with acute exacerbation.

#### *Candida* species isolation and identification

Sputum samples, deep pharyngeal swabs (taken from patients who did not produce sputum), and bronchoalveolar lavage (BAL) samples were collected and transported immediately to Microbiology Laboratory, and immediately cultured on Sabouraud dextrose agar plates with chloramphenicol (SDAC) (Difco, USA) and chromogenic agar *Candida* plates (Oxoid Ltd, UK) to isolate yeasts and to ensure purity of the isolates as reported in our previous study [14].

As no validated criteria are available, for the definition of persistent *C. dubliniensis* colonization, we based the definition on previous studies on CF airway colonization by the presence of two or more positive cultures of *C. dubliniensis* in a given year as defined in *Aspergillus fumigatus* chronic colonization [16, 17].

#### MALDI-TOF mass spectrometry

Each clinical isolate of *Candida* sp. was maintained on GYPA plates (2% glucose, 0.5% yeast extract, 1% peptone, 1.5% agar) for 48 h at 30 °C. A single colony was isolated and subcultured on SDA plates for 24 h at 30 °C. Isolates were identified by MALDI-TOF MS carried out according to the Bruker Daltonics protocol, as reported previously [18]. To ensure reproducibility of the spectra tested isolates were measured in duplicate and identified by MALDI Biotyper RTC software 3.0 (Bruker Daltonics, Germany).

#### Identification of co-existing bacteria

A routine procedure was the identification of bacterial organisms accompanying the *Candida* spp. in the lower airways of CF patients. The organisms were cultivated on a variety of different media (Remel, Lenexa, KS, USA), including trypticase soy agar with 5% sheep blood, chocolate agar, MacConkey agar, mannitol salt agar, and *B. cepacia*-selective agar. Plates were incubated in ambient air or 5% CO_2_ at 35 °C for 48 h. After Gram staining, bacteria were further identified using catalase and oxidase tests. One single colony was directly deposited on a MALDI-TOF MS identifications were performed as described previously [18].

#### Pulmonary function testing

Patient’s spirometry tests were performed routinely in each outpatient and inpatient visits in the respiratory laboratory unit in accordance with standards of the American Thoracic Society [19]. The highest of three technically appropriate measurements was recorded. Forced expiratory volume in 1 s (FEV1; in liters) was measured using a flow-sensing spirometer (Sensor Medicus Model V6200, Germany) and presented as a percent of the predicted value for children and adults.

#### Sample size

As per our review literature, probably there is no precise and accurate estimation available particularly in this region on the epidemiology of persistence of *C. dubliniensis* in the lower respiratory samples of pediatric and adult CF patients and their relationship to BMI and FEV1. Therefore, there was no formal sample size calculation done in this study. However, looking at current study designed as an observational study to address the above objectives, a total of 52 CF patients. It is worth to note that the number of available population of CF patients is very low in this small country.

#### Statistical analysis

Descriptive statistics were used to characterize the study participants in our analysis. Categorical data was expressed as a frequency along with percentage and continuous data values presented in mean ± SD and median and range. Associations between two or more qualitative variables were assessed using Chi square (χ^2^) test, and Fisher Exact test or Yates corrected Chi square as appropriate. Quantitative data between the two independent groups were analyzed using unpaired t test and Mann–Whitney U test as applicable. Repeated measure analysis of variance (ANOVA) was applied to assess the difference in BMI and FEV1% over different time points (baseline to 14 months). And when the repeated-measures ANOVA was significant (P < 0.05), we performed post hoc tests with the Bonferroni multiple pair-wise comparison method. Pictorial presentations of the key results were made using appropriate statistical graphs. All P values presented were two-tailed, and P values of <0.05 were considered as statistically significant. All statistical analyses were done using statistical packages SPSS 22.0 (SPSS Inc. Chicago, IL) and Epi-info (Centers for Disease Control and Prevention, Atlanta, GA) software.

### Results

In this study, a total of 137 respiratory samples (81 sputa, 53 deep pharyngeal swabs, and 3 BAL) were collected from 52 CF patients (pediatrics n = 38; adults n = 14). This includes 102 lower respiratory samples from pediatric and 35 lower respiratory samples from adult CF patients. Respiratory samples were obtained from outpatient clinics (84.61%) and from inpatients suffering acute CF pulmonary exacerbation (15.39%). The median number of respiratory samples per individuals was 3 (range 2–5). *C. dubliniensis* was the most prevalent *Candida* sp. 50/77 (65%) followed by *C. albicans* 21/77 (27.2%), *C. tropicalis* 5/77 (6.5%) and *C. glabrata* 1/77 (1.3%).

The mean age of 13.5 ± 8.1 years ranging from 1 to 38 years. There were 56.2% (77/137) of respiratory specimens positive for C*andida* spp. that includes (54 lower respiratory samples from pediatric and 23 lower respiratory samples from adult CF patients). Most patients were pediatrics n = 38 (73%), and male were dominant. Forty-four of CF patients with CFTR I1234 V gene mutation (30 pediatrics and 14 adults), mostly associated with pancreatic sufficiency. Fourteen pediatrics and 11 adults CF patients received anti-*Pseudomonas* nebulized tobramycin antibiotics. Oral zithromax was used in 1 pediatric and 8 adult patients. CF-related diabetes was found in 1 (2.6%) pediatric and 4 (28.6%) adult CF patients (Table 1).

**Table 1:** Summary of clinical characteristics between two CF groups ≤18 years and >18 years

Parameters	≤18 years (N = 38)	>18 years (N = 14)	P
	N(%)	N(%)	
Sex/male	21 (55.3)	9 (64.3)	0.559
No. of CF patients with CFTR 1234 V mutation	30 (78.9)	14 (100%)	0.062
No. of CF patients with other CFTR mutation	8 (21.1)	0	0.130
No. of CF patients with pancreatic sufficiency	30 (78.9)	12 (85.7)	0.583
No. of CF patients with CF related diabetes	1 (2.6)	4 (28.6)	0.005
Nebulized tobramycin use	14 (36.8)	11 (78.6)	0.008
Oral zithromax use	1 (2.6)	8 (57.1)	<0.001
Nebulized pulmozyme use	24 (63.2)	13 (92.9)	0.036
Nebulized hypertonic saline use	9 (23.7)	7 (50)	0.142

The clinical characteristics of 29 CF patients with *C. dubliniensis* isolated from sputum specimens are shown in Table 2, this includes 18 pediatric, and 11 adult CF patients were all with CFTR I1234 V mutation. Among those patients with *C. dubliniensis,* seven (38.9%) pediatrics and 8 (72.7%) adults were received anti-*Pseudomonas* nebulized tobramycin antibiotic. Oral zithromax was used only in 6 (54.5%) adult CF patients (Table 2). The persistent *C. dubliniensis* were isolated from 11/29 (37.9%) CF patients (3 pediatrics and 8 adults). Whereas, intermittent *C. dubliniensis* were isolated from 18/29 (62.1%) CF patients, in which *C. dubliniensis* was isolated only once in 13 patients, and in 5 CF patients, *C. dubliniensis* were recovered from the first sputum sample followed by other *Candida* spp. in the second sputum sample (4 CF patients had *C. albicans* in the subsequent sputum sample, and the fifth had *C. tropicalis*). Bacterial isolates were co-existed with persistent *C. dubliniensis*; in pediatric with *Staphylococcus aureus* and *Pseudomonas aeruginosa*, whereas, only *P. aeruginosa* were cultured from adult CF patients.

**Table 2:** Summary of clinical characteristics between 2 CF groups age ≤18 years and >18 years with *Candida dubliniensis*

Parameters	≤18 years (N = 18)	>18 years (N = 11)	P
	N(%)	N(%)	
Sex/male	10 (55.6)	7 (63.6)	0.668
No. of CF patients with CFTR 1234 V mutation	18 (100)	11 (100)	
No. of CF patients with pancreatic sufficiency	18 (100)	10 (90.9)	0.193
No. of CF patients with CF related diabetes	0 (0)	4 (36.4)	0.014
Nebulized Tobramycin use	7 (38.9)	8 (72.7)	0.077
Oral zythromax use	0 (0)	6 (54.5)	0.001
Nebulized pulmozyme use	12 (66.7)	10 (90.9)	0.139
Nebulized hypertonic saline use	5 (27.8)	6 (54.5)	0.149

**Fig. 1:**
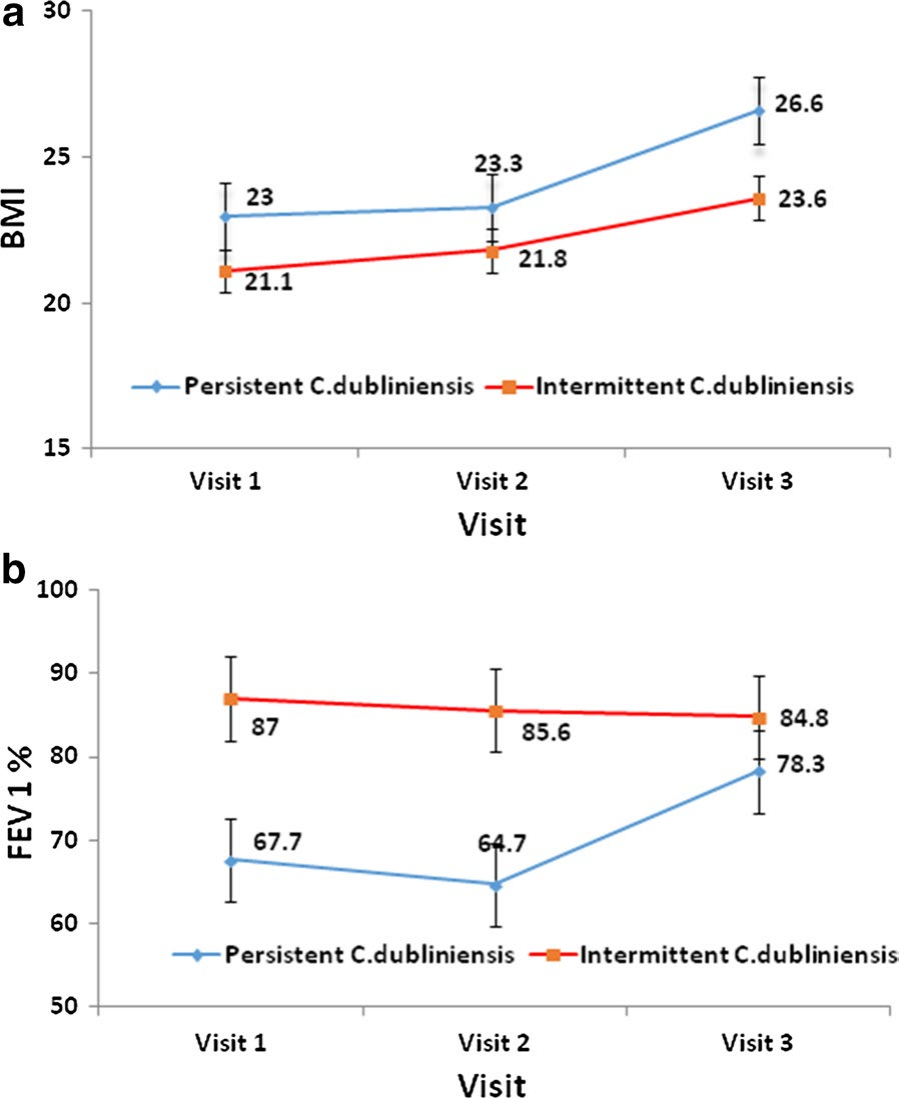
**a** The mean BMI of CF patients with the persistence and intermittent existence of *C. dubliniensis* during the study period. **b** The mean FEV_1_ of CF patients with the persistence and intermittent existence of *C. dubliniensis* during the study period

CF patients with persistent *C. dubliniensis* isolates have higher mean BMI in comparison to intermittent *C. dubliniensis* group, however, this difference did not reach statistical significance (P = 0.539) (Fig. 1a). In contrast, CF patients with persistent *C. dubliniensis* isolates have significantly lower FEV1% mean in comparison to intermittent *C. dubliniensis* group particularly at initial two visits (P < 0.05) (at 3–4 months’ interval between the two visits); however, at subsequent visit the difference observed was not statistically significant (P = 0.456), (Fig. 1b). None of the CF patients received oral or inhaled steroids or antifungal therapy.

### Discussion

Although bacteria classically dominate CF lung disease, fungal isolates are increasingly described. The prevalence of fungi in CF respiratory cultures has been reported over the last decade [20].

The prevalence rate of emerging *C. dubliniensis* pathogen is increasing and ranged from 3.8 to 39%, possibly because of advance diagnostic techniques, and improving methods for the detection of yeasts, which has been increasing in the last few years. [6, 10, 21–23]. In our previous study, we reported the highest rate of *C. dubliniensis* isolated from respiratory samples in both adult and pediatric CF patients, followed by *C. albicans*, which was explained by increased adherence properties and possibly an environmental exposure to *C. dubliniensis* [14].

A high-calorie diet has been a standard of care in CF patients for >3 decades. Higher BMI is associated with improvements in lung function in FEV1 [24]. In another study [25], reported a significant difference between malnourished and not malnourished patients with respect to FEV1%. Moreover, the patients with malnutrition were significantly more frequently colonized by *P. aeruginosa* and fungi and less so by methicillin susceptible *S. aureus*. In the present study, CF patients with persistent *C. dubliniensis* having more advanced disease, co-infections with chronic mucoid *P. aeruginosa*, CF related diabetes, long-term nebulized tobramycin and oral Zithromax therapy. In addition, they have a lower FEV1 percentage, and higher BMI than CF patients with intermittent *C. dubliniensis*. The possible explanation of higher BMI is that majority of CF patients having pancreatic sufficiency with regular follow-up in CF clinics by the dietitian. There were no significant changes observed during the 14-month follow-up regarding the FEV1 and BMI in CF patients with persistent *C. dubliniensis* and the impact on lung function and BMI needs further evaluation with long-term follow up.

Further studies are warranted to investigate if persistence of *C. dubliniensis* in the CF lung over the years is associated with chronic infection and inflammation especially if co-exist with common CF bacterial infections.

## Limitations

The study has several limitations, the low number of CF population will not capture all cases with persistent *C. dubliniensis*, the relatively short prospective follow-up study may cause some difficulties in determining the clinical significance of the course of the disease and the effect on lung function. Despite these limitations, we believe that this study is the compiled and detailed addressing of the existence of *C. dubliniensis* in respiratory secretions of CF patients.
